# Cinnamyl 2-oxo-2*H*-chromene-3-carboxyl­ate

**DOI:** 10.1107/S1600536809045644

**Published:** 2009-11-04

**Authors:** Cui-Lian Xu, Nan Yang, Guo-Yu Yang, Su-Fang Fan, Cao-Yuan Niu

**Affiliations:** a College of Sciences, Henan Agricultural University, Zhengzhou 450002, People’s Republic of China; b College of Tobacco, Henan Agricultural University, Zhengzhou 450002, People’s Republic of China

## Abstract

The title compound, C_19_H_14_O_4_, was prepared by the reaction of 2-oxo-2*H*-chromene-3-acyl chloride with cinnamic alcohol. The whole mol­ecule is not planar, the dihedral angle between the planes of coumarin and benzene rings being 13.94 (4)°, but the plane of the coumarin ring and that of the ester group are almost coplanar, making a dihedral angle of 2.9 (1)°. In the crystal structure, weak inter­molecular C—H⋯O hydrogen bonds link two mol­ecules into dimers, and π–π stacking inter­actions between inversion-related rings of the coumarin groups [centroid–centroid distance 3.8380 (15) Å with a slippage of 1.535 Å], which connect the dimers into columns extending along [010].

## Related literature

For the medicinal and biological activity of coumarins and their derivatives, see: Borges *et al.* (2005 ▶); Kontogiorgis & Hadjipavlou-Litina (2005 ▶); Gursoy & Karali (2003 ▶). For the development of coumarin derivatives as anti-HIV agents, see: Yu *et al.* (2003 ▶, 2007 ▶). For the structure of menthyl 2-oxo-2*H*-chromene-3-carboxyl­ate, see: Xu *et al.* (2009 ▶).
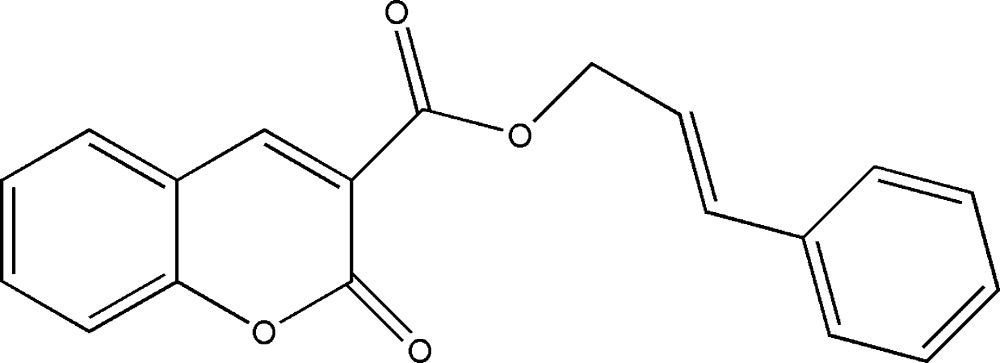


## Experimental

### 

#### Crystal data

C_19_H_14_O_4_
*M* *_r_* = 306.30
Monoclinic, 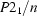

*a* = 5.7026 (11) Å
*b* = 8.2969 (17) Å
*c* = 31.693 (6) Å
β = 92.96 (3)°
*V* = 1497.5 (5) Å^3^
*Z* = 4
Mo *K*α radiation
μ = 0.10 mm^−1^
*T* = 291 K
0.20 × 0.18 × 0.18 mm

#### Data collection

Rigaku R-AXIS-IV diffractometer
Absorption correction: multi-scan (*ABSCOR*; Higashi, 1995 ▶) *T*_min_ = 0.981, *T*_max_ = 0.983
4266 measured reflections
2485 independent reflections
2002 reflections with *I* > 2σ(*I*)
*R*_int_ = 0.060

#### Refinement

*R*[*F* ^2^ > 2σ(*F* ^2^)] = 0.058
*wR*(*F* ^2^) = 0.147
*S* = 1.08
2485 reflections
209 parameters
H-atom parameters constrained
Δρ_max_ = 0.24 e Å^−3^
Δρ_min_ = −0.23 e Å^−3^

### 

Data collection: *R-AXIS* (Rigaku, 1997 ▶); cell refinement: *R-AXIS* data reduction: *R-AXIS*; program(s) used to solve structure: *SHELXS97* (Sheldrick, 2008 ▶); program(s) used to refine structure: *SHELXL97* (Sheldrick, 2008 ▶); molecular graphics: *PLATON* (Spek, 2009 ▶) and *DIAMOND* (Brandenburg, 2005 ▶); software used to prepare material for publication: *TEXSAN* (Molecular Structure Corporation & Rigaku, 2000 ▶).

## Supplementary Material

Crystal structure: contains datablocks I, global. DOI: 10.1107/S1600536809045644/si2212sup1.cif

Structure factors: contains datablocks I. DOI: 10.1107/S1600536809045644/si2212Isup2.hkl

Additional supplementary materials:  crystallographic information; 3D view; checkCIF report

## Figures and Tables

**Table 1:** Hydrogen-bond geometry (Å, °)

*D*—H⋯*A*	*D*—H	H⋯*A*	*D*⋯*A*	*D*—H⋯*A*
C5—H5*A*⋯O3^i^	0.93	2.54	3.344 (3)	145
C7—H7*A*⋯O3^i^	0.93	2.46	3.292 (3)	149

Symmetry code: (i) 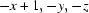.

**Fig. 1.**
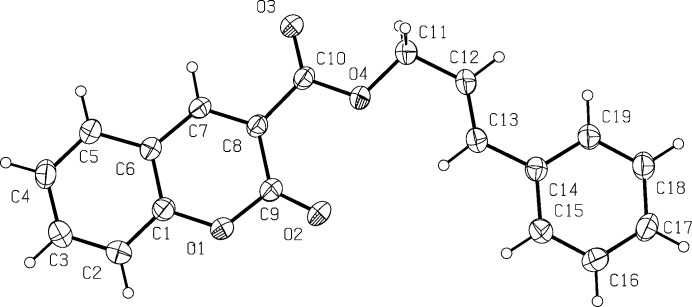
PLATON plot of (I) showing the atom numbering scheme. Displacement ellipsoids are drawn at the 30% probability level.

**Fig. 2.**
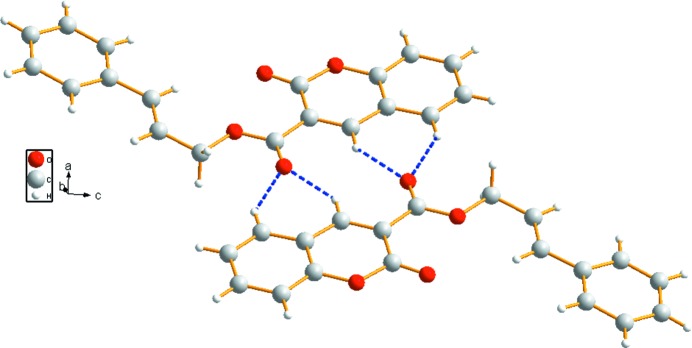
Part of the crystal structure of the title compound showing weak C—H···O hydrogen bonds as dashed lines.

**Fig. 3.**
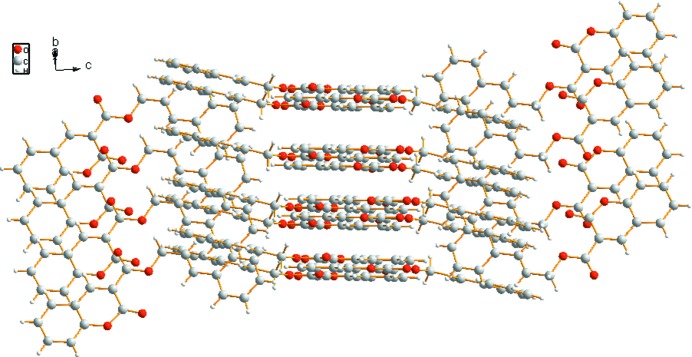
Packing diagram of the title compound.

## supplementary crystallographic information

### Comment

The coumarins and derivatives display a wide range of biological activities, such as antiviral effect (Borges *et al.*, 2005), anti-inflammatories (Kontogiorgis & Hadjipavlou-Litina, 2005), anti-bacterials (Gursoy & Karali, 2003), and anti-proliferative properties. (Yu *et al.*, 2003; Yu *et al.*, 2007), as well as being a kind of basic flavor compounds. As part of work, we have synthesized the title compound (I) and report its crystal structure here.

The molecular structure of (I) is shown in Fig. 1. It crystallizes in the E conformation, with an C11—C12—C13—C14 torsion angle of -19.6 (3)°. The plane of the coumarin ring and that of the ester group are almost co-planar, with a small dihedral angle of 2.9 (1) °, but the coumarin ring is not coplanar with the C14-benzene ring, forming a dihedral angle of 13.94 (4)°.

There are weak intermolecular C—H···O hydrogen bonds (Table 1) that link two molecules into a dimer (Fig. 2), and π-π stackings between two parallel rings [*Cg*1:O1, C1, C6, C7, C8, C9 and *Cg*2:C1^i^ - C6^i^. Symmetry code:(i) -*x*, 1 - *y*, -*z*] with a slippage of 1.535 Å and *Cg*1···*Cg*2 distance of 3.8380 (15) Å that helps to connect dimers into columns along the *b* axis (Fig. 3). The perpendicular distance between the stacked coumarin rings is 3.518 Å.

### Experimental

Compound (I) was synthesized as reported by Xu *et al.* (2009), starting from 2-oxo-2*H*-chromene-3-acyl chloride and cinnamic alcohol in equimolar amounts. Single crystals of the title compound suitable for X-ray diffractions were obtained by slow evaporation of a mixed solvent (ethyl acetate: petroleum ether = 1: 3, 7 ml) solution of the title compound (0.030 g).

### Refinement

All H atoms were placed in calculated positions, with C—H= 0.93 Å, and *U*_iso_(H)=1.2*U*_eq_(C) for aromatic and vinyl H atoms; C—H=0.97 Å, and *U*_iso_(H)=1.2 *U*_eq_(C) for methylene H atoms. The final difference map had a highest peak at 0.64 Å from atom C8 and a deepest hole at 0.95 Å from atom C9, but were otherwise featureless.

### Crystal data

**Table:**

C_19_H_14_O_4_	*F*(000) = 640
*M**_r_* = 306.30	*D*_x_ = 1.359 Mg m^−^^3^
Monoclinic, *P*2_1_/*n*	Mo *K*α radiation, λ = 0.71073 Å
Hall symbol: -P 2yn	Cell parameters from 378 reflections
*a* = 5.7026 (11) Å	θ = 1.3–25.0°
*b* = 8.2969 (17) Å	µ = 0.10 mm^−^^1^
*c* = 31.693 (6) Å	*T* = 291 K
β = 92.96 (3)°	Block, colourless
*V* = 1497.5 (5) Å^3^	0.20 × 0.18 × 0.18 mm
*Z* = 4	

### Data collection

**Table:**

Rigaku R-AXIS-IV diffractometer	2485 independent reflections
Radiation source: fine-focus sealed tube	2002 reflections with *I* > 2σ(*I*)
graphite	*R*_int_ = 0.060
Detector resolution: 0 pixels mm^-1^	θ_max_ = 25.0°, θ_min_ = 1.3°
Oscillation frames scans	*h* = 0→6
Absorption correction: multi-scan (*ABSCOR*; Higashi, 1995)	*k* = −9→9
*T*_min_ = 0.981, *T*_max_ = 0.983	*l* = −37→37
4266 measured reflections	

### Refinement

**Table:**

Refinement on *F*^2^	Secondary atom site location: difference Fourier map
Least-squares matrix: full	Hydrogen site location: inferred from neighbouring sites
*R*[*F*^2^ > 2σ(*F*^2^)] = 0.058	H-atom parameters constrained
*wR*(*F*^2^) = 0.147	*w* = 1/[σ^2^(*F*_o_^2^) + (0.0708*P*)^2^ + 0.2535*P*] where *P* = (*F*_o_^2^ + 2*F*_c_^2^)/3
*S* = 1.08	(Δ/σ)_max_ < 0.001
2485 reflections	Δρ_max_ = 0.24 e Å^−^^3^
209 parameters	Δρ_min_ = −0.22 e Å^−^^3^
0 restraints	Extinction correction: *SHELXL97* (Sheldrick, 2008), Fc^*^=kFc[1+0.001xFc^2^λ^3^/sin(2θ)]^-1/4^
Primary atom site location: structure-invariant direct methods	Extinction coefficient: 0.044 (4)

### Special details

**Table:**

Geometry. All e.s.d.'s (except the e.s.d. in the dihedral angle between two l.s. planes) are estimated using the full covariance matrix. The cell e.s.d.'s are taken into account individually in the estimation of e.s.d.'s in distances, angles and torsion angles; correlations between e.s.d.'s in cell parameters are only used when they are defined by crystal symmetry. An approximate (isotropic) treatment of cell e.s.d.'s is used for estimating e.s.d.'s involving l.s. planes.
Refinement. Refinement of *F*^2^ against ALL reflections. The weighted *R*-factor *wR* and goodness of fit *S* are based on *F*^2^, conventional *R*-factors *R* are based on *F*, with *F* set to zero for negative *F*^2^. The threshold expression of *F*^2^ > σ(*F*^2^) is used only for calculating *R*-factors(gt) *etc*. and is not relevant to the choice of reflections for refinement. *R*-factors based on *F*^2^ are statistically about twice as large as those based on *F*, and *R*- factors based on ALL data will be even larger.

### Fractional atomic coordinates and isotropic or equivalent isotropic displacement parameters (Å^2^)

**Table:**

	*x*	*y*	*z*	*U*_iso_*/*U*_eq_	
O1	−0.2090 (3)	0.32886 (19)	0.02988 (4)	0.0567 (4)	
O2	−0.1217 (3)	0.2749 (2)	0.09679 (5)	0.0696 (5)	
O3	0.4778 (3)	0.0109 (2)	0.06191 (4)	0.0638 (5)	
O4	0.2725 (3)	0.09462 (19)	0.11589 (4)	0.0534 (4)	
C1	−0.1652 (4)	0.3240 (3)	−0.01256 (6)	0.0470 (5)	
C2	−0.3243 (4)	0.4004 (3)	−0.04038 (7)	0.0621 (6)	
H2A	−0.4552	0.4528	−0.0306	0.075*	
C3	−0.2838 (5)	0.3967 (3)	−0.08278 (7)	0.0638 (7)	
H3A	−0.3886	0.4483	−0.1018	0.077*	
C4	−0.0916 (4)	0.3184 (3)	−0.09790 (7)	0.0623 (7)	
H4A	−0.0674	0.3179	−0.1267	0.075*	
C5	0.0644 (4)	0.2410 (3)	−0.07000 (6)	0.0557 (6)	
H5A	0.1928	0.1867	−0.0801	0.067*	
C6	0.0297 (3)	0.2440 (2)	−0.02644 (6)	0.0438 (5)	
C7	0.1821 (4)	0.1682 (3)	0.00463 (6)	0.0445 (5)	
H7A	0.3137	0.1139	−0.0041	0.053*	
C8	0.1425 (3)	0.1722 (2)	0.04622 (6)	0.0421 (5)	
C9	−0.0628 (4)	0.2578 (3)	0.06115 (6)	0.0493 (5)	
C10	0.3154 (4)	0.0854 (3)	0.07502 (6)	0.0451 (5)	
C11	0.4328 (4)	0.0061 (3)	0.14417 (6)	0.0581 (6)	
H11A	0.5829	0.0613	0.1468	0.070*	
H11B	0.4586	−0.1009	0.1329	0.070*	
C12	0.3306 (4)	−0.0062 (3)	0.18634 (6)	0.0532 (6)	
H12A	0.4147	−0.0667	0.2067	0.064*	
C13	0.1334 (4)	0.0596 (3)	0.19788 (6)	0.0483 (5)	
H13A	0.0479	0.1190	0.1775	0.058*	
C14	0.0349 (4)	0.0481 (2)	0.24006 (6)	0.0448 (5)	
C15	−0.1666 (4)	0.1337 (3)	0.24838 (7)	0.0557 (6)	
H15A	−0.2359	0.1990	0.2274	0.067*	
C16	−0.2664 (4)	0.1240 (3)	0.28701 (7)	0.0643 (7)	
H16A	−0.4015	0.1826	0.2918	0.077*	
C17	−0.1661 (4)	0.0276 (3)	0.31857 (7)	0.0610 (7)	
H17A	−0.2350	0.0190	0.3444	0.073*	
C18	0.0363 (5)	−0.0556 (3)	0.31136 (7)	0.0592 (6)	
H18A	0.1065	−0.1187	0.3327	0.071*	
C19	0.1365 (4)	−0.0463 (3)	0.27266 (6)	0.0530 (6)	
H19A	0.2733	−0.1036	0.2682	0.064*	

### Atomic displacement parameters (Å^2^)

**Table:**

	*U*^11^	*U*^22^	*U*^33^	*U*^12^	*U*^13^	*U*^23^
O1	0.0587 (9)	0.0672 (10)	0.0450 (8)	0.0205 (8)	0.0100 (7)	−0.0005 (7)
O2	0.0735 (11)	0.0936 (13)	0.0434 (8)	0.0265 (10)	0.0188 (8)	−0.0040 (8)
O3	0.0654 (10)	0.0836 (12)	0.0431 (8)	0.0282 (9)	0.0102 (7)	−0.0002 (8)
O4	0.0606 (9)	0.0657 (10)	0.0341 (7)	0.0156 (8)	0.0053 (6)	−0.0006 (7)
C1	0.0493 (12)	0.0461 (12)	0.0459 (11)	0.0017 (10)	0.0064 (9)	0.0010 (9)
C2	0.0594 (14)	0.0684 (16)	0.0585 (14)	0.0179 (12)	0.0030 (11)	0.0045 (12)
C3	0.0639 (15)	0.0732 (16)	0.0535 (13)	0.0069 (13)	−0.0047 (11)	0.0118 (12)
C4	0.0654 (15)	0.0820 (18)	0.0397 (12)	−0.0056 (13)	0.0043 (10)	0.0094 (11)
C5	0.0543 (13)	0.0722 (16)	0.0412 (11)	0.0038 (11)	0.0089 (10)	0.0023 (11)
C6	0.0452 (12)	0.0473 (11)	0.0394 (10)	−0.0008 (9)	0.0069 (9)	0.0009 (9)
C7	0.0449 (11)	0.0473 (12)	0.0421 (11)	0.0058 (9)	0.0098 (8)	−0.0009 (9)
C8	0.0484 (11)	0.0415 (11)	0.0370 (10)	0.0023 (9)	0.0078 (8)	−0.0018 (8)
C9	0.0527 (13)	0.0529 (13)	0.0428 (11)	0.0059 (10)	0.0085 (9)	−0.0026 (10)
C10	0.0494 (12)	0.0493 (12)	0.0374 (10)	0.0022 (10)	0.0092 (9)	−0.0028 (9)
C11	0.0596 (14)	0.0747 (15)	0.0396 (11)	0.0136 (12)	−0.0006 (10)	−0.0011 (11)
C12	0.0599 (14)	0.0633 (14)	0.0360 (10)	0.0072 (11)	−0.0022 (9)	0.0020 (10)
C13	0.0534 (13)	0.0513 (12)	0.0398 (10)	0.0007 (10)	−0.0027 (9)	0.0028 (9)
C14	0.0460 (12)	0.0469 (11)	0.0412 (10)	−0.0055 (9)	−0.0008 (9)	−0.0016 (9)
C15	0.0502 (12)	0.0658 (14)	0.0505 (12)	0.0030 (11)	−0.0015 (10)	0.0049 (11)
C16	0.0529 (14)	0.0815 (18)	0.0593 (14)	0.0091 (13)	0.0096 (11)	−0.0045 (13)
C17	0.0665 (15)	0.0762 (17)	0.0413 (12)	−0.0027 (13)	0.0130 (10)	−0.0016 (11)
C18	0.0770 (16)	0.0593 (14)	0.0410 (11)	0.0070 (13)	0.0004 (10)	0.0015 (10)
C19	0.0620 (14)	0.0541 (13)	0.0429 (11)	0.0086 (11)	0.0017 (10)	0.0006 (10)

### Geometric parameters (Å, °)

**Table:**

O1—C1	1.381 (2)	C8—C10	1.494 (3)
O1—C9	1.393 (3)	C11—C12	1.489 (3)
O2—C9	1.203 (2)	C11—H11A	0.9700
O3—C10	1.205 (2)	C11—H11B	0.9700
O4—C10	1.333 (2)	C12—C13	1.319 (3)
O4—C11	1.447 (3)	C12—H12A	0.9300
C1—C6	1.385 (3)	C13—C14	1.480 (3)
C1—C2	1.386 (3)	C13—H13A	0.9300
C2—C3	1.375 (3)	C14—C15	1.388 (3)
C2—H2A	0.9300	C14—C19	1.398 (3)
C3—C4	1.381 (4)	C15—C16	1.379 (3)
C3—H3A	0.9300	C15—H15A	0.9300
C4—C5	1.380 (3)	C16—C17	1.382 (3)
C4—H4A	0.9300	C16—H16A	0.9300
C5—C6	1.405 (3)	C17—C18	1.374 (4)
C5—H5A	0.9300	C17—H17A	0.9300
C6—C7	1.426 (3)	C18—C19	1.382 (3)
C7—C8	1.349 (3)	C18—H18A	0.9300
C7—H7A	0.9300	C19—H19A	0.9300
C8—C9	1.469 (3)		
			
C1—O1—C9	123.31 (16)	O4—C10—C8	114.69 (17)
C10—O4—C11	115.52 (16)	O4—C11—C12	109.07 (18)
O1—C1—C6	120.76 (18)	O4—C11—H11A	109.9
O1—C1—C2	117.44 (19)	C12—C11—H11A	109.9
C6—C1—C2	121.8 (2)	O4—C11—H11B	109.9
C3—C2—C1	118.3 (2)	C12—C11—H11B	109.9
C3—C2—H2A	120.9	H11A—C11—H11B	108.3
C1—C2—H2A	120.9	C13—C12—C11	126.8 (2)
C2—C3—C4	121.7 (2)	C13—C12—H12A	116.6
C2—C3—H3A	119.1	C11—C12—H12A	116.6
C4—C3—H3A	119.1	C12—C13—C14	126.4 (2)
C5—C4—C3	119.6 (2)	C12—C13—H13A	116.8
C5—C4—H4A	120.2	C14—C13—H13A	116.8
C3—C4—H4A	120.2	C15—C14—C19	117.52 (19)
C4—C5—C6	120.2 (2)	C15—C14—C13	119.69 (19)
C4—C5—H5A	119.9	C19—C14—C13	122.79 (19)
C6—C5—H5A	119.9	C16—C15—C14	121.5 (2)
C1—C6—C5	118.45 (19)	C16—C15—H15A	119.2
C1—C6—C7	117.54 (18)	C14—C15—H15A	119.2
C5—C6—C7	124.00 (19)	C15—C16—C17	120.1 (2)
C8—C7—C6	122.51 (18)	C15—C16—H16A	119.9
C8—C7—H7A	118.7	C17—C16—H16A	119.9
C6—C7—H7A	118.7	C18—C17—C16	119.4 (2)
C7—C8—C9	120.19 (18)	C18—C17—H17A	120.3
C7—C8—C10	116.55 (18)	C16—C17—H17A	120.3
C9—C8—C10	123.26 (17)	C17—C18—C19	120.6 (2)
O2—C9—O1	115.64 (19)	C17—C18—H18A	119.7
O2—C9—C8	128.7 (2)	C19—C18—H18A	119.7
O1—C9—C8	115.68 (17)	C18—C19—C14	120.8 (2)
O3—C10—O4	123.20 (19)	C18—C19—H19A	119.6
O3—C10—C8	122.10 (18)	C14—C19—H19A	119.6
			
C9—O1—C1—C6	0.9 (3)	C7—C8—C9—O1	1.1 (3)
C9—O1—C1—C2	−179.7 (2)	C10—C8—C9—O1	−178.51 (18)
O1—C1—C2—C3	−180.0 (2)	C11—O4—C10—O3	−1.0 (3)
C6—C1—C2—C3	−0.5 (4)	C11—O4—C10—C8	177.69 (18)
C1—C2—C3—C4	0.5 (4)	C7—C8—C10—O3	−2.4 (3)
C2—C3—C4—C5	0.2 (4)	C9—C8—C10—O3	177.2 (2)
C3—C4—C5—C6	−1.0 (4)	C7—C8—C10—O4	178.87 (18)
O1—C1—C6—C5	179.21 (19)	C9—C8—C10—O4	−1.5 (3)
C2—C1—C6—C5	−0.2 (3)	C10—O4—C11—C12	−166.07 (19)
O1—C1—C6—C7	−0.2 (3)	O4—C11—C12—C13	−3.8 (3)
C2—C1—C6—C7	−179.6 (2)	C11—C12—C13—C14	−179.2 (2)
C4—C5—C6—C1	1.0 (3)	C12—C13—C14—C15	175.4 (2)
C4—C5—C6—C7	−179.7 (2)	C12—C13—C14—C19	−4.5 (3)
C1—C6—C7—C8	−0.1 (3)	C19—C14—C15—C16	−1.2 (3)
C5—C6—C7—C8	−179.4 (2)	C13—C14—C15—C16	178.8 (2)
C6—C7—C8—C9	−0.4 (3)	C14—C15—C16—C17	0.0 (4)
C6—C7—C8—C10	179.19 (19)	C15—C16—C17—C18	1.4 (4)
C1—O1—C9—O2	178.6 (2)	C16—C17—C18—C19	−1.5 (4)
C1—O1—C9—C8	−1.4 (3)	C17—C18—C19—C14	0.2 (4)
C7—C8—C9—O2	−178.9 (2)	C15—C14—C19—C18	1.1 (3)
C10—C8—C9—O2	1.5 (4)	C13—C14—C19—C18	−178.9 (2)

### Hydrogen-bond geometry (Å, °)

**Table:**

*D*—H···*A*	*D*—H	H···*A*	*D*···*A*	*D*—H···*A*
C5—H5A···O3^i^	0.93	2.54	3.344 (3)	145
C7—H7A···O3^i^	0.93	2.46	3.292 (3)	149

Symmetry codes: (i) −*x*+1, −*y*, −*z*.
